# SARS-CoV-2: an Emerging Coronavirus that Causes a Global Threat

**DOI:** 10.7150/ijbs.45053

**Published:** 2020-03-15

**Authors:** Jun Zheng

**Affiliations:** 1 Faculty of Health Sciences, University of Macau, Macau SAR, China.; 2 Institute of Translational Medicine, University of Macau, Macau SAR, China.

**Keywords:** Coronavirus, Novel coronavirus, pneumonia, SARS-CoV-2, COVID-19

## Abstract

An ongoing outbreak of pneumonia caused by a novel coronavirus, currently designated as the severe acute respiratory syndrome coronavirus-2 (SARS-CoV-2), was reported recently. However, as SARS-CoV-2 is an emerging virus, we know little about it. In this review, we summarize the key events occurred during the early stage of SARS-CoV-2 outbreak, the basic characteristics of the pathogen, the signs and symptoms of the infected patients as well as the possible transmission pathways of the virus. Furthermore, we also review the current knowledge on the origin and evolution of the SARS-CoV-2. We highlight bats as the potential natural reservoir and pangolins as the possible intermediate host of the virus, but their roles are waiting for further investigation. Finally, the advances in the development of chemotherapeutic options are also briefly summarized.

## Introduction

**Figure 1:**
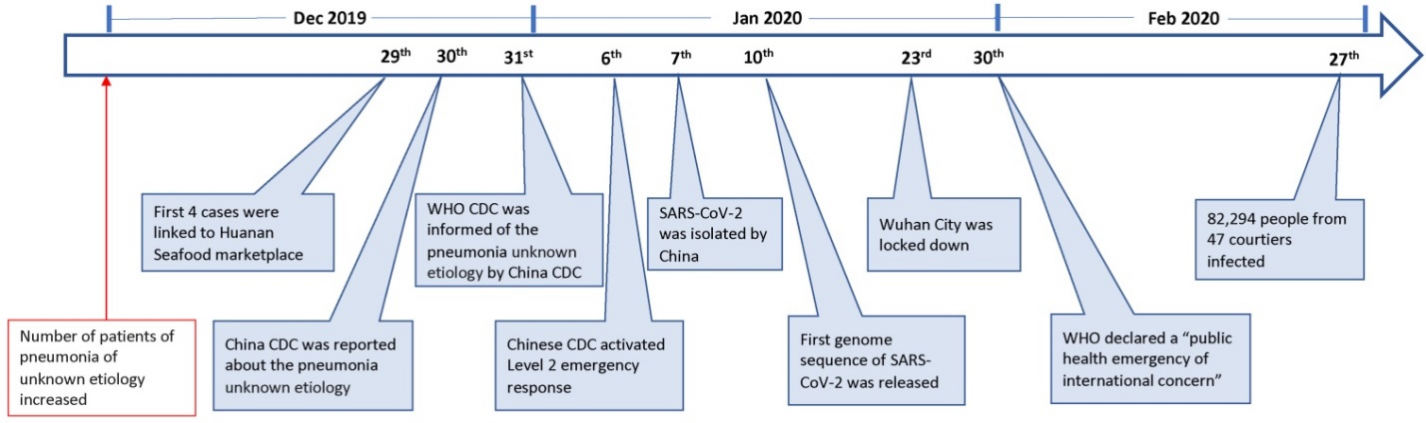
Key events in the early stage of SARS-CoV-2 outbreak.

On 23 Feb 2020, the lock-down of Wuhan, a central city in China, has alarmed people all over the world of an emerging novel coronavirus that is posing a major public health and governance challenges. The novel virus, previously called the 2019-novel coronavirus (2019-nCoV), is currently designated as the severe acute respiratory syndrome coronavirus-2 (SARS-CoV-2). As of 27 Feb, this emerging infection has been reported in 47 countries, causing over 82,294 infections with 2,804 deaths (Fig. 1) 1. This novel virus is also becoming a mounting threat to Chinese and global economies.

Coronaviruses (CoVs) are members of the family Coronaviridae, the enveloped viruses that possess extraordinarily large single-stranded RNA genomes ranging from 26 to 32 kilobases in length 2. CoVs have been identified in both avian hosts and various mammals, including bat, camels, dogs and masked palm civets, and are previously regarded as pathogens that only cause mild diseases in the immunocompetent people until the emergence of the coronavirus causing severe acute respiratory syndrome (SARS-CoV) in late of 2002 3-6. Currently, at least seven coronavirus species are known to cause diseases in humans. The viruses of 229E, OC43, NL63 and HKU1 only cause common cold symptoms, which are mild. Severe illness can be caused by the remaining three viruses, namely SARS-CoV, which resulted in the outbreak of SARS in 2002 and 2003 3,4; the coronaviruses that are responsible the Middle East respiratory syndrome (MERS-CoV), which emerged in 2012 and remains in the circulation in camels 7; and SARS-CoV-2, the viruses emerged in December 2019 in Wuhan of China and a great effort is being undertaken to contain its spreading 8. In this review, we will briefly introduce the outbreak history of SARS-CoV-2, the signs and symptoms of the infected patients, its transmission dynamics, the advances in the understanding on its evolutional origin and the chemotherapeutic options being developed for the treatment of its infection.

### The key events of SARS-CoV-2 outbreak and the pathogen characteristics

Since December 2019, an increasing number of patients with pneumonia of unknown etiology in Wuhan, a city with 11 million people, have alarmed the local hospital. On 29 December 4 cases were linked to Huanan Seafood wholesale market 9, where non-aquatic live animals, including several kinds of wild animals, were also on the sales. The local Center for Disease Control (CDC) then found additional patients linked to the same market after investigation, and reported to China CDC on 30 Dec 2019 9. The second day, World Health Organization (WHO) was informed of the cases of pneumonia of unknown etiology by China CDC 10. On 6 Jan 2020, a level 2 emergency response was launched by China CDC 11.

The causal agent was not identified until 7 Jan 2020; a new type of coronavirus was isolated by Chinese authority 10. The genome sequence of SARS-CoV-2 (WH-Human_1) was first released and shared by China on 10 Jan 12. The isolation and identification of SARS-CoV-2 apparently facilitated the development of molecular diagnostic methods and the confirmation of the infected patients. As of 21 Jan, there are 270 cases were confirmed from Wuhan 13. On 23 Jan, Wuhan city was locked down by local government. On 30 Jan, WHO declared a “public health emergency of international concern” (Fig. 1).

Subsequently, the viruses were successfully isolated from several laboratories 8,14,15. The virion of SARS-CoV-2 looks like a solar corona by transmission electron microscopy imaging: the virus particle is in a spherical shape with some pleomorphism; the diameter of the virus particles range from 60 to 140 nm with distinctive spikes about 8 to 12 nm in length 8. The observed morphology of SARS-CoV-2 is consistent with the typical characteristics of the Coronaviridae family. The genome sequence of SARS-CoV-2 from clinical samples has been obtained by several laboratories with deep sequencing 8,14-18. The viral genome of SARS-CoV-2 is around 29.8 kilobase, with a G+C content of 38%, in total consisting of six major open reading frames (ORFs) common to coronaviruses and a number of other accessory genes 14,16. The sequences analysis showed that the genome sequences of viruses from different patients are very conserved 14,15,19, implying that the human virus evolves recently.

### Signs and symptoms of patients infected by SARS-CoV-2

A typical characteristic of the SARS-CoV-2 infected patient is pneumonia, now termed as Coronavirus Disease 2019 (COVID-19), demonstrated by computer tomographic (CT) scan or chest X -ray 3,8,18. In the early stages, the patients showed the acute respiratory infection symptoms, with some that quickly developed acute respiratory failure and other serious complications 20. The first three patients reported by the China Novel Coronavirus Investigating and Research Team all developed severe pneumonia and two of these three patients with available clinical profiles showed a common feature of fever and cough 8. A subsequent investigation of a family of six patients in the University of Hong Kong-Shenzhen Hospital demonstrated that all of them had pulmonary infiltrates, with a variety of other symptoms 18. The chest X-ray and CT imaging in a study showed that 75% of 99 patients demonstrated bilateral pneumonia and the remaining 25% unilateral pneumonia 21. Overall, 14% of the patients showed multiple mottling and ground-glass opacity 21. The first cases of coronavirus infection in the United States also showed basilar streaky opacities in both lungs by chest radiography. However, the pneumonia for this patient was only detected on the day 10 of his illness 22. It is also of note that one of patients among the family of six patients did not present any other symptoms and signs, but had ground-glass lung opacities identified by CT scan 18.

At least four comprehensive studies on the epidemiological and clinical characteristics of SARS-CoV-2 infected patients have been performed 21,23-25. The most common signs and symptoms of patients are fever and cough 21,23-25. Fatigue was complained by 96% of patients (n=138) in one study 24, but was less outstanding (18%, n=44) in another report 23. A combinational analysis of the common recorded signs or symptoms of the reported cases found that fever was observed in around 90% of the SARS-CoV-2 infected patients; the number of patients with cough is relatively less (68%) compared to fever (Table 1). In addition, shortness of breath or dyspnea, muscle ache, headache, chest pain, diarrhea, haemoptysis, sputum production, rhinorrhoea, nausea and vomiting, sore throat, confusion, and anorexia were also observed in a proportion of the patients 21,23-25 (Table 1).

A common feature of patients of SARS, MERS or COVID-19 is the presence of severe acute respiratory syndrome; however, the estimated fatality rate of COVID-19 (2.3%) is much lower than SARS (~10%) and MERS (~36%) 26,27. Furthermore, the viruses responsible for above three diseases are evolutionary distinct (See below for details) 19.

### Transmission of the virus

It is clear now that SARS-CoV-2 can be transmitted by human-to-human despite the majority of the early cases had contact history with the Huanan Seafood market 11,18,28. Analysis of 425 patients with confirmed COVID-19 showed that the incubation period is 3 to 7 days. The mean was 5.2 days (95% CI: 4.1 to 7.0), and the 95^th^ percentile of the distribution is 12.5 days (95% CI: 9.2 to 18) 11. Notably, it was reported that the incubation period could be as long as 24 days in a rare case 25. The basic reproductive number (R_0_) up to the period of 4 Jan 2020 was estimated based on the study of 425 patients to be 2.2 (meaning that one patient has been spreading infection to 2.2 other people) 11, slightly smaller than the value of 2.68 by a modelling in another 29. The R_0_ of SARS-CoV-2 from both of these two studies is smaller than that of SRAS, which are 3 before public health measures were implemented 30. However, subsequent investigation based on the analysis of high-resolution real-time human travel and infection data estimated that the R_0_ is much larger, ranging from 4.7 to 6.6 before the control measures 31, implying that SARS-CoV-2 is highly contagious and more infectious than initially estimated. This conclusion is consistent with the wide spread of SARS-CoV-2 within a short period time and was also echoed by the finding that SARS-CoV-2 Spike (S) protein had 10- to 20-fold higher affinity to human angiotensin-converting enzyme 2 (ACE2) receptor than that of SARS-CoV based on the Cryo-EM structure analysis of S proteins 32. Similar to SARS-CoV, the entry of SARS-CoV-2 into host cells depends on the recognition and binding of S protein to ACE2 receptor of the host cells 14,33. The high affinity of S protein to ACE2 receptor likely contributes to the quick spreading of virus. The finding of ACE2 as the receptor of SARS-CoV-2 also indicates that human organs with high ACE2 expression level, such as lung alveolar epithelial cells and enterocytes of the small intestine, are potentially the target of SARS-CoV-2 34.

As a new coronavirus, it is not known yet about how SARS-CoV-2 spreads. Current knowledge for SARS-CoV-2 transmission is largely based on what is known from the similar coronaviruses, particularly SARS-CoV and MERS-CoV, in which human-to-human transmission occurs through droplets, contact and fomites. SARS-CoV is predominantly transmitted through indirect or direct contact with mucous membranes in the mouth, eyes, or nose 35. It has been shown that unprotected eyes and exposed mucous membranes are vulnerable to SARS-CoV transmission 36. A member of the national expert panel on pneumonia was infected by SARS-CoV-2 after the inspection in Wuhan 37. As he wore a N95 mask but not any eye protector, and experienced eye redness before the onset of pneumonia, it was thus suspected that unprotected exposure of the eyes to SARS-CoV-2 might be another transmission pathway 37. However, SARS-CoV-2 was not detected from the conjunctival swab sample in a confirmed COVID-19 patent with conjunctivitis 38, suggesting that more evidences are needed before concluding the conjunctival route as the transmission pathway of SARS-CoV-2. The mode of transmission by MERS-CoV is not well understood but is believed to spread largely via the respiratory close contact route 39,40.

Based on the transmission mode of SARS-CoV and MERS-CoV, a serial of preventive measures have been recommended, including avoiding close contact with people suffering from acute respiratory infections and frequent hand-washing 41. The viruses of SARS-CoV-2 were also detected in the stool samples in some patients but not all 18,22, suggesting that a possible fecal-oral transmission occurs 42. A systematic study showed that viruses could be detected in oral swabs, anal swabs and blood samples of the patients, and the anal swabs and blood could test positive when oral swab tested negative 43. Furthermore, a trend of shift from more oral positive in the collected samples during the early period of patient infection to more anal positive during later period of infection was also found 43. Therefore, a multiple shedding routes of SARS-CoV-2 might exist.

One of the challenges for preventive control of SARS-CoV-2 spreading is that the viruses are likely transmitted by asymptomatic contact. A German businessman was found infected by SARS-CoV-2 after attending a conference together with a colleague, who had no signs or symptoms of infection but had become ill due to the SARS-CoV-2 infection later 44. This observation suggests that infected patients likely start to shed viruses before the onset of any symptom, which undoubtedly will bring great challenge to the current practice of preventive control by measuring body temperature. Despite the claim of the transmission by asymptomatic contact has been challenged 45, other asymptomatic carriers were also observed to transmit the viruses of SARS-CoV-2 46,47. Consistently, a study found that an asymptomatic patient had a similar vial loads in the samples of nasal and throat swabs to that of the symptomatic patients 48.

### The origin and evolution of SARS-CoV-2

It is critical to identify the origin, native host(s) and evolution pathway of the virus that causes an outbreak of a pandemic. This information can help understand the molecular mechanism of its cross-species spread and implement a proper control measure to prevent it from further spreading. The association of initially confirmed SARS-CoV-2 cases with Huanan Seafood market suggested that the marketplace has played a role in the early spreading 11,23, however, whether it is the origin of the outbreak and what is the native host(s) of SARS-CoV-2 remain uncertain. In fact, the firstly documented patient was not linked to Huanan Seafood market 23.

The analysis of SARS-CoV-2 origin was firstly performed based on the genome sequence of virus isolates from six patients 19. When compared with SARS-CoV and MERS-CoV, the nucleotide sequences of SARS-CoV-2 showed a higher homology with that of SARS-CoV while was relatively poor with that of MERS-CoV 19. Despite some of the six major OFRs of SARS-CoV-2 genes share less than 80% identity in nucleotide acids to SARS-CoV, the seven conserved replicase domains in ORF1ab has 94.6% sequence identity in amino acids between SARS-CoV-2 and SARS-CoV 14, suggesting that these two viruses might belong to the same species. The origin of SARS-CoV has been extensively investigated. Masked palm civets were initially considered to transmit SARS-CoV to humans as a close variant of SARS-CoV was detected from palm civets 49. This conclusion was supported by the fact that three of the four patients had the record of contact with palm civets during the two small-scale of SARS outbreaks occurred in late 2003 and early 2004 50, 51. However, a deep investigation based on the genome sequence of isolated viruses showed that SARS-CoV-like virus in civet had not been circulating for long 52. Subsequently, coronaviruses with high similarity to the human SARS-CoV or civet SARS-CoV-like virus were isolated from horseshoe bats, concluding the bats as the potential natural reservoir of SARS-CoV whereas masked palm civets are the intermediate host 53-56.

It is thus reasonable to suspect that bat is the natural host of SARS-CoV-2 considering its similarity with SARS-CoV. The phylogenetic analysis of SARS-CoV-2 against a collection of coronavirus sequences from various sources found that SARS-CoV-2 belonged to the *Betacoronavirus* genera and was closer to SARS-like coronavirus in bat 19. By analyzing genome sequence of SARS-CoV-2, it was found that SARS-CoV-2 felled within the subgenus *Sarbecovirus* of the genus Betacoronavirus and was closely related to two bat-derived SARS-like coronaviruses, bat-SL-CoVZC45 and bat-SL-CoVZXC21, but were relatively distant from SARS-CoV 15, 18, 57-59. Meanwhile, Zhou and colleagues showed that SARS-CoV-2 had 96.2% overall genome sequence identity throughout the genome to BatCoV RaTG13, a bat coronavirus detected in *Rhinolophus affinis* from Yunnan province 14. Furthermore, the phylogenetic analysis of full-length genome, the receptor binding protein spike (S) gene, and RNA-dependent RNA polymerase (RdRp) gene respectively all demonstrated that RaTG13 was the closest relative of the SARS-CoV-2 14. However, despite SARS-CoV-2 showed high similarity to coronavirus from bat, SARS-CoV-2 changed topological position within the subgenus *Sarbecovirus* when different gene was used for phylogenetic analysis: SARS-CoV-2 was closer to bat-SL-CoVZC45 in the S gene phylogeny but felled in a basal position within the subgenus *Sarbecovirus* in the ORF1b tree 57. This finding implies a possible recombination event in this group of viruses. Of note, the receptor-binding domain of SARS-CoV-2 demonstrates a similar structure to that of SARS-CoV by homology modelling but a few variations in the key residues exist at amino acid level 15, 19.

Despite current evidences are pointing to the evolutional origin of SARS-CoV-2 from bat virus 15,57, an intermediate host between bats and human might exist. Lu et. al. raised four reasons for such speculation 15: First, most bat species in Wuhan are hibernating in late December; Second, no bats in Huanan Seafood market were sold or found; Third, the sequence identity between SARS-CoV-2 and bat-SL-CoVZC45 or bat-SL-CoVZXC21, the closest relatives in their analyses, is lower than 90%; Fourth, there is an intermediate host for other human-infecting coronaviruses that origin from bat. For example, masked palm civet and dromedary camels are the intermediate hosts for SARS-CoV 49 and MERS-CoV respectively 60. A study of the relative synonymous codon usage (RSCU) found that SARS-CoV-2, bat-SL-CoVZC45, and snakes had similar synonymous codon usage bias, and speculated that snake might be the intermediate host 61. However, no SARS-CoV-2 has been isolated from snake yet.

Pangolin was later found to be a potential intermediate host for SARS-CoV-2. The analysis of samples from Malytan pangolins obtained during anti-smuggling operations from Guangdong and Guangxi Customs of China respectively found novel coronaviruses representing two sub-lineages related to SARS-CoV-2 62. The similarity of SARS-CoV-2 to these identified coronaviruses from pangolins is approximately 85.5% to 92.4% in genomes, lower than that to the bat coronavirus RaTG13 (96.2%) 14,62. However, the receptor-binding domain of S protein from one sub-lineage of the pangolin coronaviruses shows 97.4% similarity in amino acid sequences to that of SARS-CoV-2, even higher than that to RaTG13 (89.2%) 62. Interestingly, the pangolin coronavirus and SARS-CoV-2 share identical amino acids at the five critical residues of RBD of S protein, while RaTG13 only possesses one 62. The discovery of coronavirus close to SARS-CoV-2 from pangolin suggests that pangolin is a potential intermediate host. However, the roles of bat and pangolin as respective natural reservoir and intermediate host still need further investigation.

### Chemotherapeutic options for SARS-CoV-2 infection

As an emerging virus, there is no effective drug or vaccine approved for the treatment of SARS-CoV-2 infection yet. Currently, supportive care is provided to the patients, including oxygen therapy, antibiotic treatment, and antifungal treatment, extra-corporeal membrane oxygenation (ECMO) etc. 21,22. To search for an antiviral drug effective in treating SARS-CoV-2 infection, Wang and colleagues evaluated seven drugs, namely, ribavirin, penciclovir, nitazoxanide, nafamostat, chloroquine, remdesivir (GS-5734) and favipiravir (T-750) against the infection of SARS-CoV-2 on Vero E6 cells *in vitro* 63. Among these seven drugs, chloroquine and remdesivir demonstrated the most powerful antiviral activities with low cytotoxicity. The effective concentration (EC_50_) for chloroquine and remdesivir were 0.77µM and 1.13µM respectively. Chloroquine functions at both viral entry and post-entry stages of the SARS-CoV-2 infection in Vero E6 cells whereas remdesivir does at post-entry stage only. Chloroquine is a drug used for an autoimmune disease and malarial infection with potential broad-spectrum antiviral activities 64,65. An EC_90_ (6.90 µM) against the SARS-CoV-2 in Vero E6 cells is clinically achievable *in vivo* according to a previous clinical trial 66. Remdesivir is a drug currently under the development for Ebola virus infection and is effective to a broad range of viruses including SARS-CoV and MERS-CoV 67,68. Functioning as an adenosine analogue targeting RdRp, Remdesivir can result in premature termination during the virus transcription 69,70. The EC_90_ of remdesivir against SARS-CoV-2 in Vero E6 cells is 1.76 µM, which is achievable *in vivo* based on a trial in nonhuman primate experiment 63,69. Encouragingly, in the first case of SARS-CoV-2 infection in the United States, treatment with remdesivir was provided intravenously to the patient on the day 7 without any adverse events observed. The patient's clinical condition was improved on day 8 and the previous bilateral lower-lobe rales disappeared, implying the remdesivir might be effective to the treatment of SARS-CoV-2 infection 22. This result, however, should be interpreted with caution as this is only single case study and a proper trial control was lacking. In addition, baricitinib, a Janus kinase inhibitor, was also predicted to reduce the ability of virus to infect lung cell by an analysis of BenevolentAI 71.

Currently, chloroquine and remdesivir are under phase 3 clinical trial and open-label trial for treatment of SARS-CoV-2 infection respectively (Table 2) 72. Preliminary results showed that chloroquine phosphate had apparent efficacy in treatment of COVID-19 73. However, caution must be taken during clinical use of chloroquine as its overdose is highly fatal without known antidote 74. Despite the lack of documented *in vitro* data supporting the antiviral efficacy on SARS-CoV-2, several antiviral chemotherapeutic agents have been registered for the clinical trials for the treatment of COVID-19 (Table 2) 72.

### Conclusion remarks

SARS-CoV-2 is an emerging pathogen, without any effective drug available for treatment at the moment. It spreads quickly and can result in death of the infected patients. Despite the current mortality rate is 2.3% 26, the emergence of large number of infected patients within short period of time could result in the collapse of health care system, and thus the mortality rate might be elevated. Effective preventive measures must be implemented to control it from global spreading. In addition, great effort should be made on the development of vaccine and antiviral drugs. Meanwhile, the intermediate host and the molecular mechanism of its cross-species spread should be further investigated. Legislation should be employed to prohibit the trade of wild animals, the potential intermediate host(s) of various viruses, to prevent the outbreak of this and other novel viruses in future.

## Figures and Tables

**Table 1:** Common signs and symptoms of SARS-CoV-2 infected patients from four reports

Signs or Symptoms	Number of patients with signs or symptoms from each report				Number of patients with signs or symptoms	Total number of patients	Percentage
	Report 1 21	Report 2 23	Report 3 24	Report 4 25			
Fever	82 (n=99)	40 (n=41)	136 (n=138)	975 (n=1099)	1233	1377	90%
Cough	81 (n=99)	31 (n=41)	82 (n=138)	745 (n=1099)	939	1377	68%
Sputum production/Expectoration	NR	11 (n=39)	37 (n=138)	370 (n=1099)	418	1276	33%
Shortness of breath/Dyspnoea	31 (n=99)	22 (n=40)	43 (n=138)	205 (n=1099)	301	1376	22%
Headache	8 (n=99)	3 (n=38)	9 (n=138)	150 (n=1099)	170	1374	12%
Sore throat/Pharyngalgia	5 (n=99)	NR	24 (n=138)	153 (n=1099)	182	1336	14%
Diarrhoea	2 (n=99)	1 (n=38)	14 (n=138)	42 (n=1099)	59	1374	4%

NR: Not Recorded.

**Table 2:** Summary of chemotherapeutic drugs under clinical trial for COVID-19

Name of Drug	Target and Mode of Action in other Viruses	*In Vitro* Antiviral Activity to SARS-CoV-2	Clinical Trial Status for COVID-19	Clinical Trial Registration Number
Remdesivir (GS-5734)	Inhibits RdRp 70	Tested 63	Phase 3	NCT04252664; NCT04257656
Favipiravir	Inhibits RdRp 75	Tested 63	Randomized trial	ChiCTR2000029544; ChiCTR2000029600
Ribavirin	Inhibits viral RNA synthesis and mRNA capping 76	Tested 63	Randomized trial, incombination a pegylated interferon	ChiCTR2000029387
Lopinavir	Inhibits 3C like protease (3Clpro) 77	Not tested	Phase 3	NCT04252274; NCT04251871; NCT04255017; ChiCTR2000029539
Ritonavir	Inhibits 3Clpro 77	Not tested	Phase 3	NCT04251871; NCT04255017; NCT04261270
Darunavir and Cobicistat	Inhibits HIV protease 78	Not tested	Phase 3	NCT04252274
ASC09F (HIV protease inhibitor)	Inhibits HIV protease 79	Not tested	Phase 3, in combination with oseltamivir	NCT04261270
Chloroquine	A lysosomatropic base that appears to disrupt intracellular trafficking and viral fusion events 80	Tested 63	Open-label trial	ChiCTR2000030054; ChiCTR2000029939; ChiCTR2000029935; ChiCTR2000029899; ChiCTR2000029898; ChiCTR2000029837; ChiCTR2000029803; ChiCTR2000029761; ChiCTR2000029740; ChiCTR2000029559; ChiCTR2000029542; ChiCTR2000029868; ChiCTR2000029826; ChiCTR2000029762; ChiCTR2000029760; ChiCTR2000029609
Arbidol (Umifenovir)	Block viral fusion 81	Not tested	Phase 4	NCT04260594; NCT04254874; NCT04255017
Oseltamivir	Inhibit neuaminidase 82	Not tested	Phase 3 and Phase 4	NCT04255017; NCT04261270
